# New Clue: Prediction from Cell-Free DNA

**DOI:** 10.3390/jcm9072307

**Published:** 2020-07-21

**Authors:** Yan Y. Sanders

**Affiliations:** Division of Pulmonary, Allergy and Critical Care Medicine, Department of Medicine, University of Alabama at Birmingham, Birmingham, AL 35294, USA; yans@uab.edu

**Keywords:** lung transplant, CLAD, cell-free DNA, cf-DNA, BAL, CXCL10

## Abstract

The main challenge for a positive long-term outcome in lung transplantation is the lack of early detection for chronic lung allograft dysfunction (CLAD). With advancements in technology, an increasing number of studies demonstrate that cell-free DNA (cfDNA) in body fluids could be used as a marker for disease diagnosis, prognosis or monitoring response to treatment. A previous report from this journal found the joint assessment of cfDNA and CXCL10 from brochoalveolar lavage (BAL) could determine the subphenotypes of CLAD and predict lung transplant survival. This is an exciting attempt in monitoring the progress for lung transplant recipients. More studies and better understanding of cfDNA are needed to develop an accessible and reliable biomarker to monitor the progress of CLAD to improve the long-term survival for lung transplant recipients.

Lung transplantation is the treatment option for patients with end-stage lung diseases [1]. Compared to other solid organ transplantation, the long-term outcomes of lung transplant remain poor, which is largely due to the chronic lung allograft dysfunction (CLAD) that usually develops in half of the recipients at 5 years post-lung transplant [2]. Since there is no effective treatment to reverse CLAD, it is critically important to prevent it through detection, and to create targeted strategies to slow its progress. Therefore, establishing diagnostic or prognostic biomarkers for CLAD diagnosis or prediction is crucial to improve long-term survival.

There are many risk factors for developing CLAD after lung transplant, such as acute cellular rejection, lymphocytic bronchiolitis and antibody-mediated rejection [3]. CLAD is usually diagnosed by serial transbronchial lung biopsies [3,4]. However, this diagnostic method has limited reliability, and is invasive and risky [5,6]. Therefore, brochoalveolar lavage (BAL) and plasma samples have been explored to identify biomarkers in acute rejection, but no markers have been established [6]. Less invasive biopsies, “liquid biopsies” have emerged as a critical, potentially alternative approach to tissue biopsies. Liquid biopsies usually refer to analysis of circulating nucleic acids, such as cell-free DNA (cfDNA) [7]. cfDNA are the fragmented DNA found in the body fluid that is 150–200 base pair long from nucleases digested cellular DNA whose molecular origin remains poorly understood, but likely from apoptotic cells or tissues [8]. cfDNA can be found in many tissues, including blood, which is thought that cfDNA is a reflection of a person’s health and disease [8]. Recently, donor-derived cell-free DNA (dd-cfDNA) has been used as a noninvasive diagnostic test; the measurement of dd-cfDNA as a fraction of the total cfDNA can detect rejection in heart, lung, liver and kidney allografts [9,10]. Some studies indicated that cfDNA combined with other markers could improve the diagnosis of CLAD significantly [11].

For the liquid biopsies of lung transplant, BAL from the lung transplant recipient would offer direct sampling of the cellular and molecular events from the anatomic site of lung allograft rejection. Attempts were made to identify markers from BAL fluid to detect allograft rejection [12]; however, no marker has been identified that has the predictive power [13]. From previous studies, a pro-inflammatory cytokine—CXCL10—in BAL was reported to be associated with the risk factor of CLAD development [14]. A study by Yang et al. reported using cfDNA and CXCL10 derived from BAL to detect and distinguish subphenotypes of CLAD and to predict lung transplant survival [15]. They showed CXCL10 and cfDNA together could distinguish sub-phenotypes of CLAD and segregate low- and high-survival patient populations. The study used joint assessment of CXCL10 and cfDNA in BAL to evaluate CLAD, which is innovative. Although CXCL10 from BAL has been linked with high risk of CLAD [14] and survival phenotypes of CLAD [16], cfDNA evaluation is usually obtained from blood. BAL is a widely used technique in pulmonary medicine to look into the microenvironment of the lung; usually the proteins in BAL have been investigated as biomarkers for acute rejection and CLAD in lung transplantation [17]. By analyzing the BAL, Yang et al. [15] found that cfDNA could distinguish the sub-phenotypes of CLAD, while cfDNA and CXCL10 together could provide significant survival prediction of CLAD patients. They also examined other traditionally used biomarkers in BAL, such as IL-6 and IL-8, which did not provide better projection.

The findings in this study are important as they offer new ways to establish biomarkers to monitor lung transplant recipients for developing CLAD. The survival rate in lung transplantation is about 6 years, which is behind other solid organ transplantations, and CLAD is the major impediment of survival [18]. However, the sample size in this study is small; large studies would reveal more information and a better predictive value of the lung allograft conditions by the changes in BAL. More studies are needed to apply such methods to find biomarkers for CLAD. Studies using cfDNA from blood could be used to monitor CLAD development more frequently, as BAL is still a relatively invasive procedure. This study only measured the cfDNA in the BAL, a parallel study to compare the cfDNA obtained from plasma and BAL would generate valuable information if cfDNA from plasma has similar predictive power as those from BAL, and if these cfDNA could be used to monitor the progress of transplant recipients.

In this study, cfDNA was measured by an ELISA-like kit [15]. With the advancement of technology, cfDNA can be quantified by droplet digital PCR and next-generation sequencing [19]. There are studies establishing approaches using shotgun sequencing to measure single nucleotide polymorphism differences in the donor and recipient to establish “genetic fingerprint” to monitor the progression of transplantation [20]. Such quantitative measurements, along with other biomarkers, would offer powerful surveillance for the early detection of CLAD, and improve the survival of lung transplantation recipients. There is great progress made in non-invasive methods for early detection of CLAD; studies from Yang et al. [15] provide a novel approach. Their report supports the need for more detailed, large studies to establish reliable biomarkers to monitor the development of CLAD, in order to improve the overall survival rate for lung transplant recipients.
